# Evidence of the Zanclean megaflood in the eastern Mediterranean Basin

**DOI:** 10.1038/s41598-018-19446-3

**Published:** 2018-01-18

**Authors:** Aaron Micallef, Angelo Camerlenghi, Daniel Garcia-Castellanos, Daniel Cunarro Otero, Marc-André Gutscher, Giovanni Barreca, Daniele Spatola, Lorenzo Facchin, Riccardo Geletti, Sebastian Krastel, Felix Gross, Morelia Urlaub

**Affiliations:** 1 Marine Geology & Seafloor Surveying, Department of Geosciences, University of Malta, Msida, Malta; 2 Istituto Nazionale di Oceanografia e di Geofisica Sperimentale (OGS), Trieste, Italy; 3 Instituto de Ciencias de la Tierra Jaume Almera, CSIC, Barcelona, Spain; 4 Laboratoire Géosciences Océan, University of Brest/CNRS, IUEM, Pl. N. Copernic, Plouzané, 29280 France; 5 Dipartimento di Scienze Biologiche, Geologiche e Ambientali, Università di Catania, Catania, Italy; 6 Christian-Albrechts-Universität zu Kiel, Institut für Geowissenschaften, Kiel, Germany; 7 GEOMAR Helmholtz Centre for Ocean Research Kiel, Kiel, Germany

## Abstract

The Messinian salinity crisis (MSC) - the most abrupt, global-scale environmental change since the end of the Cretaceous – is widely associated with partial desiccation of the Mediterranean Sea. A major open question is the way normal marine conditions were abruptly restored at the end of the MSC. Here we use geological and geophysical data to identify an extensive, buried and chaotic sedimentary body deposited in the western Ionian Basin after the massive Messinian salts and before the Plio-Quaternary open-marine sedimentary sequence. We show that this body is consistent with the passage of a megaflood from the western to the eastern Mediterranean Sea via a south-eastern Sicilian gateway. Our findings provide evidence for a large amplitude drawdown in the Ionian Basin during the MSC, support the scenario of a Mediterranean-wide catastrophic flood at the end of the MSC, and suggest that the identified sedimentary body is the largest known megaflood deposit on Earth.

## Introduction

The Messinian salinity crisis (MSC) was an outstanding palaeo-oceanographic event that affected the Mediterranean region from 5.97 to 5.33 Ma^1^. A temporary restriction of the Atlantic-Mediterranean seaway induced an imbalance between evaporation and water inputs^2^, transforming the Mediterranean Sea into a giant hypersaline lake and resulting in the deposition of kilometre-thick sequences of salts. A widespread interpretation involves the partial desiccation of the Mediterranean Sea during the Messinian^3^, with proposed sea level drawdowns of 1300–2400 m^4,5^. Following the sampling of MSC sedimentary sequences during the Deep Sea Drilling Project (DSDP) in the 1970s, the Zanclean megaflood hypothesis has been regarded as a plausible scenario for the termination of the MSC. However, the presence of brackish lacustrine deposits atop Messinian salts has been used to question this hypothesis, suggesting instead an overspill of Paratethyan water (former Black Sea) followed by Atlantic inflow once the Mediterranean Basin was refilled^6^. According to the catastrophic flood theory, topographic sills in the Mediterranean Basin underwent extensive erosion that should be identifiable in the sedimentary record further downstream^7,8^. However, evidence for deposition of the eroded material has so far been elusive.

## Seismic Stratigraphy Of The Western Ionian Basin

**Figure 1:**
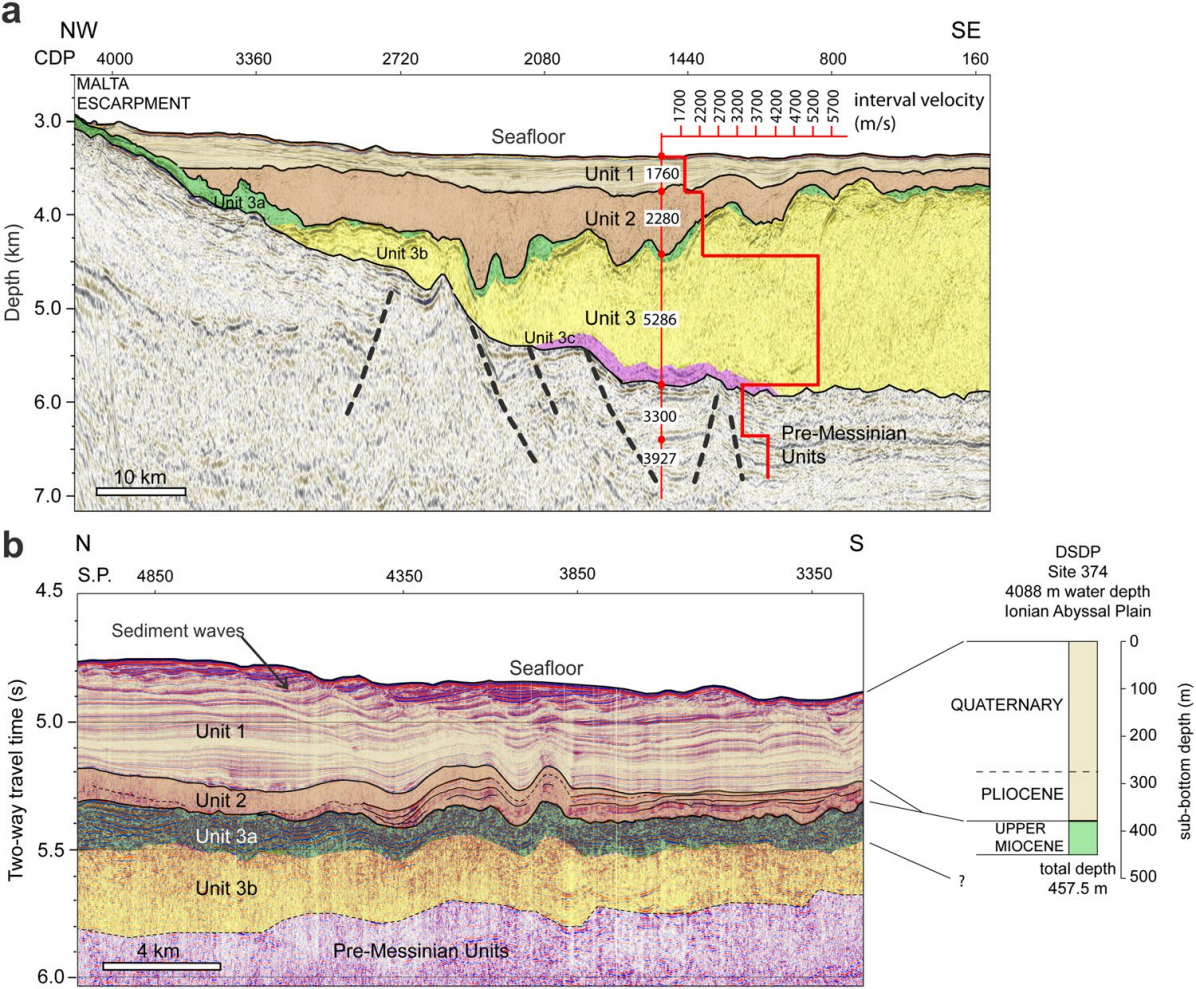
Seismic stratigraphy of western Ionian Basin. Interpreted units on (**a**) PSDM seismic reflection profile CROP 21 and (**b**) post-stack time-migrated seismic reflection profile CUMECS-3. A plot of estimated PSDM seismic velocities is included in (**a**). Location of profiles in Fig. 2a. Correlation with DSDP Site 374 (location in Fig. 2a) is provided for (**b**). Dashed black lines denote inferred faults. CDP = common depth point; SP = shot point. Unit 1 consists of high amplitude, continuous, parallel to sub-parallel reflectors, which correlate with Pliocene-Quaternary pelagics, contourites and turbidites (Supplementary Table S1). In (**b**), the shallower section of unit 1 is shaped into sediment waves. Unit 3 correlates with MSC evaporites (Supplementary Table S1) and comprises a discontinuous, highly reflective and mostly deformed upper unit (3a - gypsum and marls), an intermediate, typically reflector-less unit with a basin-fill geometry (3b - halite), and a lower, highly reflective, discontinuous unit (3c - lower gypsum or clastics). The top of unit 3 slopes towards the south-south-east. Unit 2 is described in the text. In (**b**), unit 2 is imaged at a transition from a chaotic to laminated seismic facies. The un-interpreted seismic reflection profiles are displayed in Supplementary Fig. S1.

**Figure 2:**
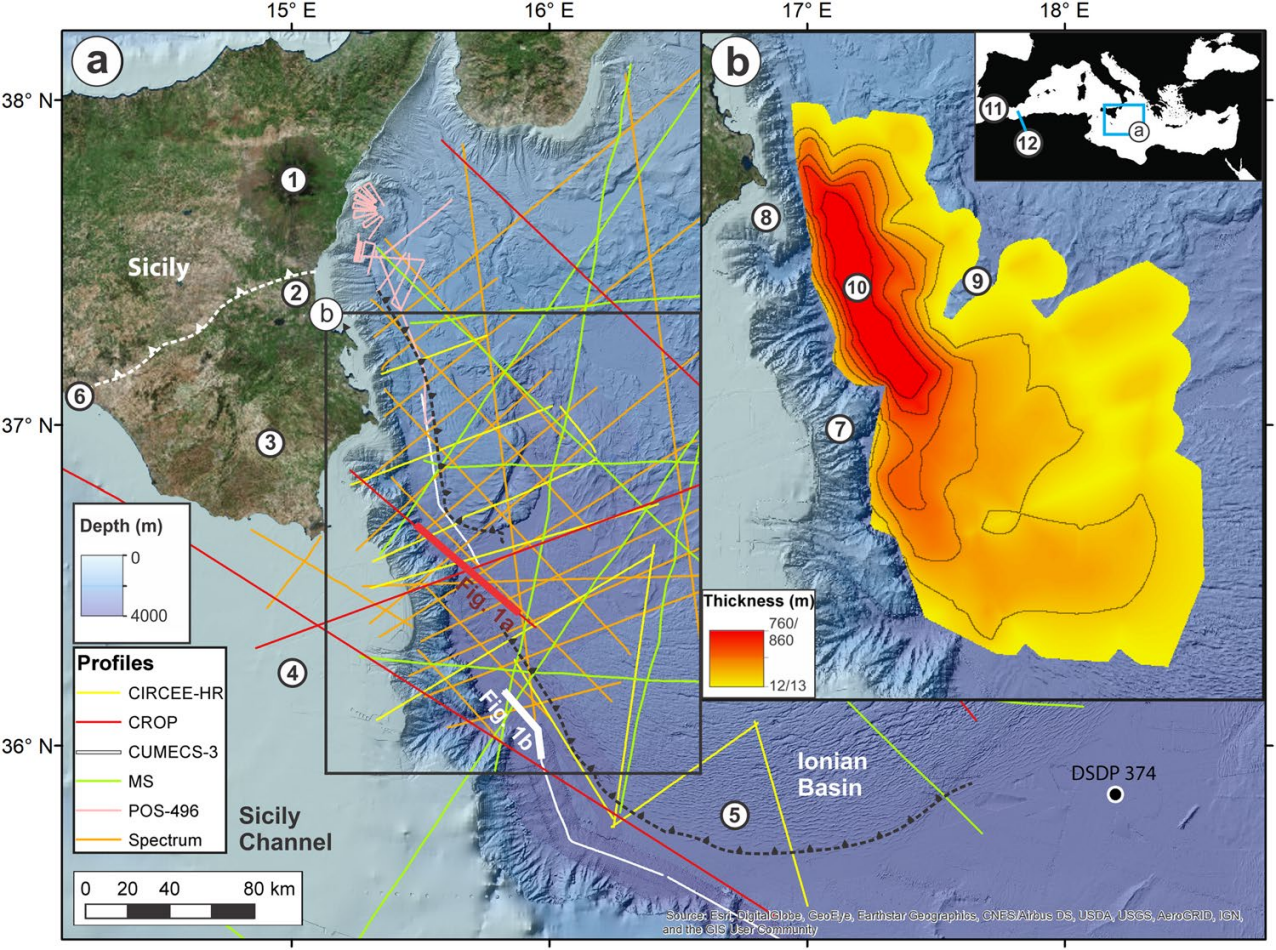
Location and geometry of unit 2. (**a**) Bathymetric map of the eastern Sicily Channel and western Ionian Basin, and location of analysed seismic reflection profiles. Location of DSDP Site 374^13^ is denoted. 1: Mt Etna; 2: Catania Plain; 3: Hyblean Plateau; 4: Malta Plateau; 5: Calabrian accretionary wedge; 6: Sicilian Fold and Thrust Belt. (**b**) Isopach map of unit 2 based on 2.3/2.6 km s^−1^ PSDM seismic velocities (contour interval of 150/172 m). 7: Malta Escarpment; 8: Noto Canyon; 9: Alfeo Seamount; 10: Depocentre of unit 2. Inset: Location of (a) in the Mediterranean Sea. 11: Straits of Gibraltar; 12: Alborán Sea. Maps generated with ArcMap 10.2 (http://www.esri.com/arcgis/). Background data from a recent compilation^39^, EMODnet bathymetry (www.emodnet-bathymetry.eu/), and a compilation of data from ESRI, DigitalGlobe, GeoEye, Earthstar Geographics, CNES Airbus DS, USDA, USGS, AeroGRID, and IGN.

The Sicily Channel was the bathymetric sill separating the western and eastern Mediterranean Basins during the Messinian^9^. The Messinian to Recent stratigraphy of the western Ionian Basin, to the east of the Sicily Channel, has been constrained using seismic reflection and DSDP borehole data^10–13^. It consists of Plio-Quaternary fine-grained marine sediments (unit 1) and Messinian evaporites (unit 3), which are locally separated by a distinct body with chaotic to transparent seismic characteristics (unit 2) (Fig. 1). We have compiled the most complete geophysical database from the western edge of the Ionian Basin to map the extent, form and seismic character of unit 2 (Fig. 2). Terminating abruptly against the Malta Escarpment to the west, unit 2 covers an area of 160 km × 95 km and has a wedge-shaped geometry that thins eastwards. It varies laterally from basin fill at the foot of the Malta Escarpment, to a drape featuring intermediate amplitude and discontinuous reflectors on the gentle folds of the outer Calabrian accretionary wedge. Using pre-stack depth migration (PSDM) seismic velocities of 2.3 and 2.6 km s^−1^, derived from seismic profiles CROP 21 and Archimede-16^10^ and typical of moderately consolidated marine sediments, we estimate that unit 2 has a maximum thickness of 760–860 m and volume of 1430–1620 km^3^.

We use morphologic and seismic stratigraphic evidence from our geophysical database to dismiss three hypotheses previously proposed for the origin of unit 2. The first is that unit 2 resulted from an extensive submarine mass movement^11^. The Malta Escarpment is not prone to large-scale slope instability, however. It consists of exposed and indurated Mesozoic to Cenozoic limestone and dolomite where evidence of rapid tectonic or sedimentary loads is lacking, at least during the last 6 Ma^14,15^. There are no visible large-scale mass movement scars on the escarpment (Fig. 2), and pronounced scar modification is unlikely in such a sediment-starved environment. Flank collapse of Mt Etna could not have generated unit 2 as this volcano is only 0.5 Ma old^16^. Unit 2 has also been interpreted as folded Messinian upper gypsum, deformed as a consequence of the south-westward growth of the Calabrian accretionary wedge^17^. Nevertheless, the measured PSDM seismic velocities (2.3–2.6 km s^−1^) are not compatible with the high velocity expected in gypsum layers (>3.1 km s^−1^)^18^. Unit 2 does not show internal structures indicative of the suggested folding, and its depocentre lies on top of the undeformed and laterally continuous upper gypsum (unit 3a in Fig. 1b). Finally, unit 2 may be part of the “Complex Unit”, described elsewhere as a deposit of material eroded from upper to lower Mediterranean continental slopes during lower sea level phases of the MSC^12,19^. However, unit 2 always occurs on top of the evaporitic sequence and does not represent a lateral transition into it. Unit 2 shows neither seaward prograding stratal configuration nor fan-shaped geometry. The cumulative volume of the canyons across the entire Malta Escarpment (1100 km^3^), which could have provided a source of material for unit 2 as a Complex Unit, is lower than the volume of unit 2. Broad and thick lenses, representative of sedimentary lobes sourced by canyon erosion on the Malta Escarpment, are only visible beneath unit 3b (halite) (Supplementary Fig. S3). There are no large fluvial drainage systems in the vicinity of unit 2 to act as a sediment source, which is generally the case for other extensive Complex Units^12^. For similar reasons, unit 2 cannot be correlated to the Reworked Lower Gypsum (RLG) and associated Mass Transport Complex (MTC) traced in the neighbouring Sirte Gulf^20^. In particular, the RLG-MTC units are located stratigraphically below the Upper Evaporites and Lago-Mare formations in shallower water depths of the upper continental margin. They are interpreted as the result of mass wasting on a continental shelf, undergoing rapid-sedimentation and over-steepening, at the mouth of the huge Eosahabi River and Neogene Lake Chad drainage system.

## The Zanclean Megaflood Hypothesis

Here we test the hypothesis that unit 2 is a deposit of material eroded and transported across the Sicily Channel once the western Mediterranean Sea level reached the sill during the Zanclean megaflood at the end of the MSC^7^. The transparent-chaotic seismic internal configuration of unit 2 indicates rapid mass deposition in the vicinity of the Malta Escarpment. Thinning and seismic lamination occurring with increasing distance towards the south-east (Fig. 1b) suggest lower-energy deposition, potentially involving finer material. According to previous models constrained with geophysical imaging of erosional features at the Strait of Gibraltar, the high flow velocities and peak discharge rates estimated at both this strait and the Sicily Sill should be similar (up to 45 m s^−1^ and 10^8^ m^3^ s^−1^, respectively)^7^. Based on modelling constrained by geological and geophysical observations, the water level in the eastern Mediterranean was up to 2400 m lower than at present^4,21^. Therefore, the head loss and the erosional features produced by the megaflood across the Sicily Sill (430 m in depth^7^) must have been comparable to those documented in the Strait of Gibraltar area (ca. 1000 km^3^ of reworked sediment)^8^. In the light of this, the material composing unit 2 is likely to have been predominantly derived from incision of the sill by eastward flowing water. In this scenario, the megaflood would have transferred water and material eroded from the sill in subaerial conditions and entered a deep brine-filled Ionian Basin at the base of the Malta Escarpment. Gypsum and halite saturation at the end of the MSC imply that seawater salinity in the Ionian Basin was about 5–10 times higher than present seawater^22^. Sediment dispersal mechanisms in such a dense saline water body involve a large fraction of clasts in suspension^23^ and would explain the lack of traction structures in the seismic record of unit 2. At the end of the MSC, accommodation space for the deposition of unit 2 was available because the western Ionian Basin was already a deep basin and the pre-Messinian Calabrian and Mediterranean deformation fronts were in a landward position with respect to the present^13,24^. The post-Messinian deformation front of the salt-bearing Calabrian accretionary wedge has subsequently propagated southwards, inducing conformable deformation of the eastern edge of unit 2 and the underlying unit 3.

The sedimentary record of the termination of the MSC in the Eastern Mediterranean as fan-shaped, fluvio-deltaic formations resting on the Messinian salt unit has been imaged in seismic reflection profiles from the Sirte Basin in the Sirte Gulf^20^ and in the Levant Basin^25–27^. In both cases, the seismic character of the formations is defined by high reflectivity, lateral discontinuity of reflectors, and a highly irregular upper boundary that includes multiple v-shaped channels. These formations are a few hundred metres thick in the Sirte Gulf as a result of the very large Eosahabi - Chad drainage system, and only a few tens of metres thick in the Levant Basin, as a result of the smaller extent of the Abu Madi, Afiq, and Nahr Menashe palaeo-drainage systems offshore Egypt, Israel and Lebanon, respectively. No such character is found in unit 2, which reinforces our interpretation that it represents a unique Zanclean megaflood deposit terminating the MSC in the western Ionian Basin. Both our interpretation of unit 2 and the fluvio-deltaic Lago-Mare formations mapped in the Eastern Mediterranean imply a significant sea level drawdown until the termination of the MSC.

**Figure 3:**
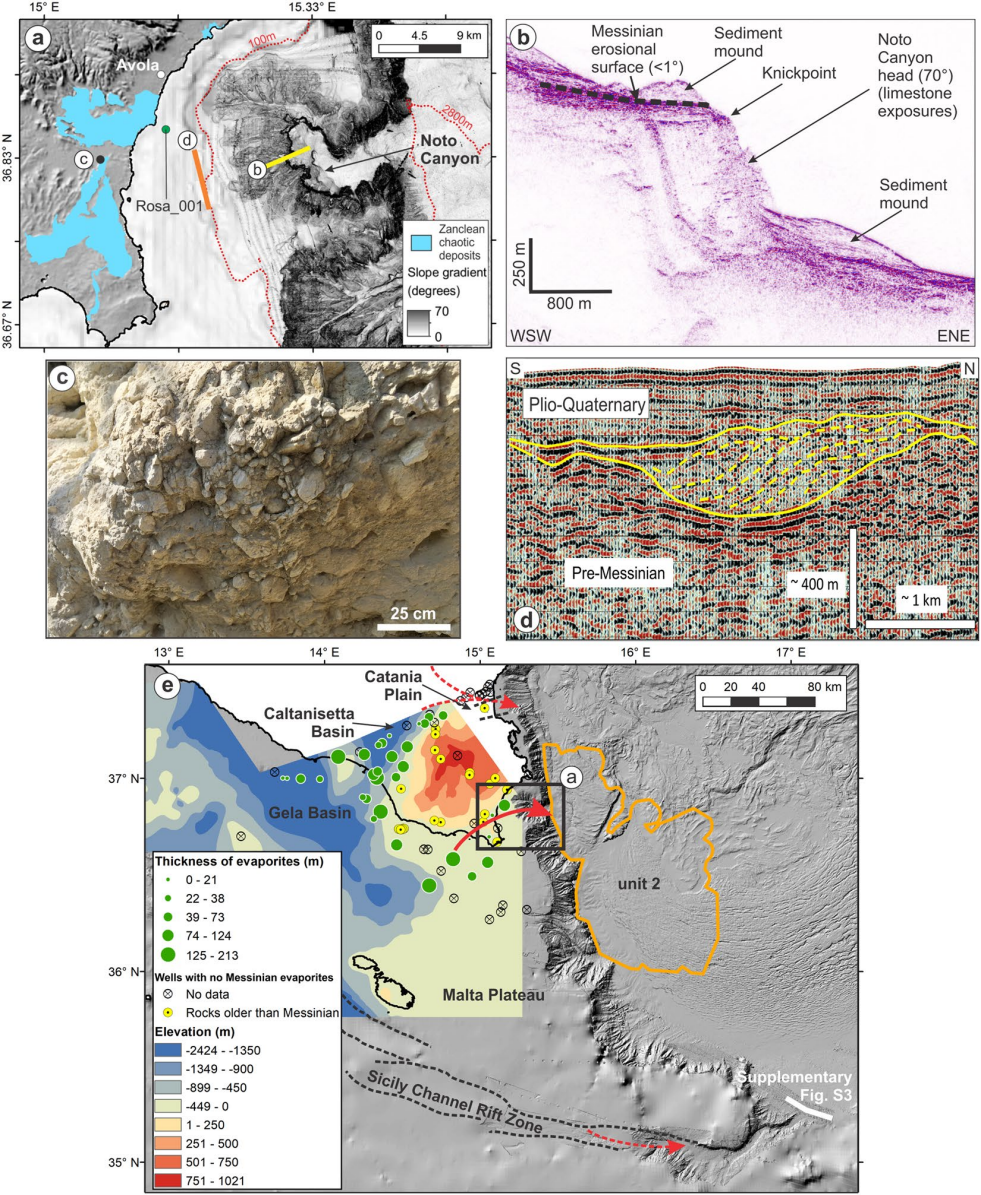
Gateway for the passage of the Zanclean flood to the eastern Mediterranean. (**a**) Shaded relief model of south-eastern Sicily showing terrestrial chaotic Zanclean deposits, and seafloor slope gradient map showing Noto Canyon. Isobaths denoted by dotted red lines. Location in (**e**). (**b**) Seismic reflection profile CIR-07 showing Noto Canyon head and Messinian erosional surface upslope. Location in (**a**). Interpretation is based on correlation with nearby wells (shown in (**e**)). (**c**) Photograph of terrestrial chaotic Zanclean deposits. Location in (**a**). (**d**) Seismic reflection profile C-578, located upslope of Noto Canyon, showing a channel eroded in pre-Messinian limestones. Channel fill is marked by large-scale downlap and sigmoidal internal configuration (denoted by yellow lines), suggesting infilling by high energy deposition, and is sealed by Plio-Quaternary muds. Interpretation and depth conversion based on well Rosa_001 (location in (**a**)). Un-interpreted profile in Supplementary Fig. S2. (e) Messinian topography (above and below present sea level) across south-east Sicily and Malta Plateau, generated using published seismic reflection profiles and well data (Supplementary Fig. S4), overlaid on shaded relief model of present bathymetry. Thickness of Messinian evaporites from well data is included. Potential flow paths of the Zanclean flood are indicated by red arrows (solid = likely; dashed = unlikely). Extent of unit 2 is outlined in orange. Dashed black lines denote boundaries of graben structures. Maps generated with ArcMap 10.2 (http://www.esri.com/arcgis/). Background data from a recent compilation^39^ and EMODnet bathymetry (www.emodnet-bathymetry.eu/).

We identify south-eastern Sicily as the most likely gateway for the eastern Mediterranean Zanclean megaflood. The depocentre of unit 2 is located in front of the mouth of Noto submarine canyon, the morphology of which is unique on the Malta Escarpment (Figs 2b, 3a). Carved in hard Mesozoic limestones, the canyon head has a slope gradient of up to 70° and relief of 700 m. These are the highest values reported from any canyon incised in carbonate margins worldwide^28^. The 6 km wide amphitheatre-shaped head of Noto Canyon is similar to that of bedrock canyons rapidly eroded by megafloods^29,30^. Upslope of Noto Canyon is an erosional channel, 4 km wide and ~400 m deep, which is infilled and buried beneath 300 m of Plio-Quaternary sediments (Fig. 3d). We thus interpret Noto Canyon as the collector of the cascading flow into the Ionian Basin, and the slope change between the steep canyon head and the <1° Messinian erosional surface upstream (Fig. 3b) as a knickpoint that retreated during the megaflood. Based on the available data, the erosional signature of the Zanclean megaflood in the Sicily Channel appears to be smaller than that in the Strait of Gibraltar; this may be a result of the more competent seabed materials being eroded in the former (shallow water carbonates) compared to the latter (flysch)^7,31,32^. The preferential accumulation of unit 2 south of Noto Canyon can be explained as a result of the inherited bathymetric surface of unit 3 and the rightwards Coriolis force exerted over the water and sediment flow during the flood.

To the south of the Hyblean Plateau, especially in the vicinity of the head of Noto Canyon, Messinian evaporites recorded in wells are thicker than those to the north (Fig. 3e). We interpret this as indicative of deeper Messinian terrains to the south of the Hyblean Plateau, which could have provided a preferential route for flood water towards the Ionian Basin. The Hyblean Plateau shows no evidence of Messinian evaporites and was always emerged during the MSC; it therefore comprised a barrier to flooding. South-east Sicily also hosts chaotic terrestrial deposits that are Zanclean in age^33^; they are composed of centimetre to decimetre-wide calcareous breccia and angular gravel and pebbles (Fig. 3a,c), suggesting high energy transport by water. The deposits lack facies typical of channel and floodplain mountain alluvium, while no palaeo-drainage systems have been identified in the south-eastern Hyblean Plateau. Additionally, the internal structure of these deposits is entirely different from that of sediments of the same age outcropping further north in the Sicilian Fold and Thrust Belt, which consist of well-bedded, fine grained carbonate marls.

Other potential gateways for the Zanclean megaflood are less plausible. The erosion of the other numerous canyons across the Malta Escarpment has been attributed to subaerial fluvial erosion before deposition of Messinian evaporites^34,35^. The Catania Plain, between Mt Etna and the Hyblean Plateau, is presently a NE-SW trending structural low that may suggest a connection between the Ionian Basin and the evaporite-bearing Messinian foredeep Caltanissetta Basin, or any other Messinian basin in northern Sicily. However, the onset of tectonic subsidence in the Catania Plain is late Pliocene in age^36^, and regional borehole data do not provide evidence of evaporites or reworked clastic deposits in this area (Fig. 3e). The 30 km wide gap with the northern limit of unit 2 is also difficult to explain. Another gateway may have been located in the graben system of the Sicily Channel Rift Zone, as present-day seafloor bathymetry suggests (Fig. 3e). However, such a scenario is not consistent with the new observations concerning the relation of unit 2 to the timing and pattern of megaflooding. The Sicily Channel Rift Zone was mainly active after the MSC^37^, whilst its eastern termination is located 60 km south of unit 2. Seismic data across the grabens do not provide evidence for large-scale erosion and transport of sediment^34,37^.

## Implications

Based on a combination of geological and geophysical data, our preferred interpretation of unit 2 is that of a body deposited by the Zanclean megaflood during its passage from the western to the eastern Mediterranean via a gateway located in south-eastern Sicily. Unit 2 is larger in volume than the material eroded by the Zanclean megaflood in the Alborán Sea^8^ and the largest terrestrial outburst floods (e.g. Missoula, Bonneville, and Altay^38^). Our findings suggest that the Zanclean megaflood was a Mediterranean-wide event, and confirm that the eastern Mediterranean Basin experienced large-scale sea level drawdown during the MSC. Detailed investigation of unit 2, especially via drilling and numerical modelling, will provide a unique opportunity to better constrain megaflood dynamics, and to address controversies related to the mechanisms of the transition to open marine conditions and sea level fluctuations during the MSC.

## Methods

### Multibeam echosounder data

Multibeam echosounder bathymetry data (Fig. 2) were acquired during the following surveys:

(i) R/V Meteor, cruise 86, 2011: PI – S. Krastel (Kongsberg Simrad EM122 and EM710);
(ii) R/V Suroît, CIRCEE-HR, 2013: PI – M.-A. Gutscher (Kongsberg Simrad EM302);
(iii) R/V OGS Explora, CUMECS-2, 2014: PI – A. Micallef (Reson SeaBat 7150 and 8111);
(iv) R/V OGS Explora, CUMECS-3, 2015: PI – A. Camerlenghi (Reson SeaBat 7150 and 8111).

Data were processed, by accounting for sound velocity variations and basic quality control, to derive grids of 10 m × 10 m to 30 m × 30 m bin sizes. Background data were provided by a recent compilation^39^ and EMODnet bathymetry (www.emodnet-bathymetry.eu/).

### Multichannel seismic reflection profiles

Multichannel seismic reflection profiles from the following surveys were used to map unit 2 (Fig. 2b):

(i) MS, 1969–1973: PI – various (acquisition and processing protocol described in^40^).
(ii) CROP, 1988–1995: PI – various (acquisition and processing protocol described in^41^). Pre-stack depth migration was conducted on profile CROP-21, as the length of the streamer allows for a reliable interval velocity determination. This involved the following procedures: trace editing, geometric corrections, water bottom picking, deghosting, velocity analysis, quality control stacking, stacking (shot interpolation, geometry update, surface-related multiple attenuation, gain correction, multichannel deconvolution, uniform offset gathers) and pre-stack depth migration (conversion of stack velocity field to interval velocity in depth, pre-stack Kirchhoff depth migration, common image gathers quality control and residual velocity analysis, interval velocity field update, final velocity field, pre stack Kirchhoff depth migration, automatic gain control removal, FX deconvolution, time variant filtering, balancing, final stacking).
(iii) CA-99, 1999: SPECTRUM. Source included an air gun array (total volume of 56 l). Receiver included a 6000 m long streamer with average hydrophone spacing of 12.5 m. Processing protocol involved filtering, signature deconvolution, velocity analysis, radon demultiple attenuation, pre-stack migration, normal move-out correction, inverse migration, bandpass filtering, post-stack scaling and minimum to zero phase conversion.
(iv) MEM-07, 2007: SPECTRUM. Source included an air gun array (total volume of 36 l). Receiver included a 7200 m long streamer with average hydrophone spacing of 12.5 m. Processing protocol involved low-cut filtering, zero phase de-signature, gain recovery, time frequency de-noising, velocity analysis, pre-stack time migration, radon demultiple attenuation, bandpass filtering and post-stack scaling.
(v) Meteor cruise 86, 2011: PI – S. Krastel (acquisition and processing protocol described in^42^).
(vi) CIRCEE-HR, 2013: PI – M.-A. Gutscher (acquisition and processing protocol described in^24^).
(vii) CUMECS-3, 2015 PI: – A. Camerlenghi. Source included one GI-Gun and one Mini GI-Gun (total volume of 4.4 l). Receiver included a 300 m long GEOMETRICS GeoEel digital streamer with average channel spacing of 3.125 m. Processing involved trace editing, geometric corrections, band-pass filtering, re-sampling, velocity analysis and stacking, post-stack spiking deconvolution, post-stack depth migration with finite difference method, and time variant filtering.
(viii) Poseidon cruise 496, 2016 PI: – S. Krastel. Source included one Sercel Mini-GI-Gun with a total volume of 0.4 l. Receiver included a GEOMETRICS GeoEel digital solid-state streamer with 80 channels and a group spacing of 1.5625 m. Processing includes band-pass filtering, geometry setup, normal move-out correction, common mid-point stacking and a finite difference migration.

### Estimation of volume of unit 2

The top and bottom of unit 2 were interpreted as horizons using the above multichannel seismic reflection profiles. The difference in time between the two horizons was calculated and converted into depth using PSDM seismic velocities of 2.3 and 2.6 km s^−1^, derived from seismic profile CROP 21 and Archimede-16, respectively^10^. An isopach map of unit 2 was generated by interpolating the depth estimations using a natural neighbour technique. The boundaries of the isopach map are restricted to the areas where unit 2 has been identified. The volume of unit 2 was estimated by multiplying the value of depth by the grid area (100 m × 100 m) and adding up all the values.

### Generation of Messinian topographic surface

To generate the Messinian surface across south-east Sicily, the Malta Plateau and Gela Basin (Fig. 3e), we used:

(i) Seismic reflection profiles (G82–032 to G82–73; G82–101 to G82–157; C-502 to C-616; C-1002 to C-1022) (spatial coverage in Supplementary Fig. S4) published in VIDEPI (http://unmig.sviluppoeconomico.gov.it/videpi/). These data were recorded using an air gun and water gun array (total volume of 32.8 l) with a 2400 m long streamer equipped with 96 groups, 15 hydrophones per group, group interval of 25 m and an active section length of 50 m.
(ii) Well data (Acate_001_Dir, Acate_002, Acate_003, Alfa_001, Archimedes_001, Aretusa_001, Biddusa_002, Capo_Negro_001, Carla_001, Cassibile_001, Cernia_001, Cianciana_001, Corvina_Mare_001, Delfino_001, Egeria_001, Eraclea_Sicilia_001, Eva_001, Gabbiano_001, Genziana_001, Genziana_001_Dir_A, Giada_001, Leone_001, Lucata_001_X, Maddalena_001, Manfria_002, Marinella_003, Marzamemi_001, Merluzzo_Mare_001, Mila_001, Mila_002, Mila_002_Bis, Mila_003, Mila_004, Mila_004_Dir, Mila_005, Mila_006, Mila_006_Dir, Mila_007_Dir, Mila_008_Dir, Mila_009_Dir, Nanda_001, Nettuno_001, Nuccia_001, Olga_001, Onda_001, Oreste_001, Orione_Est_001, Orlando_001, Orlando_002, Oscar_Ovest_001, Pachino_004, Palma_001, Palma_002, Palma_003, Pamela_001, Pamela_001_Bis, Pancrazio_Sud_001, Paola_Est_001, Patty_Est_001, Pellicano_Ovest_001, Piera_001, Pilade_Est_001, Pina_001, Plinio_Sud_001, Polpo_001, Porto_Palo_001, Rosa_001, Samanta_001, Santuzza_001, Sirio_001, Sofia_001, Spada_Mare_001, Spigola_Mare_001, Ulisse_001, Vallo_001, Venere_001, Ventura_001, Vera_001, Zagara_001) (spatial coverage in Supplementary Fig. S4) published in VIDEPI (http://unmig.sviluppoeconomico.gov.it/videpi/).

The seismic reflection profiles were digitised into SEG-Y format from.pdf images. We identified the top surface or margin erosion surface, which are seismic markers associated with the MSC, and then converted these values to depths using the well data. From the well data, we identified the depth of the top of the Messinian. This was deducted from the depth of the top of the well and referred to the present elevation or bathymetry. The Messinian topographic surface was generated using the ANUDEM elevation gridding procedure^43^.

### Estimation of submarine canyon volume along Malta Escarpment

To estimate the volume of the submarine canyons along the Malta Escarpment, we projected isobaths from adjacent un-eroded areas (e.g. canyon interfluves). We generated a surface using a linear interpolation, calculated the difference in elevation from the original bathymetry, multiplied the difference by the grid area (100 m × 100 m), and added up all the values.

### Data availability

The multibeam echosounder data, and multichannel seismic reflection profiles (from MS, CROP, Meteor 86, CIRCEE-HR, CUMECS-3 and Poseidon 496), are available from the corresponding author upon reasonable request.

The multichannel seismic reflection profiles from CA-99 and MEM-07 are available from SPECTRUM but restrictions apply to the availability of these data, which were used under license for the current study, and so are not publicly available. Data are, however, available from the authors upon reasonable request and with permission of SPECTRUM.

## Electronic supplementary material

Supplementary information
