# Supplementary material for: Evidence of the Zanclean megaflood in the eastern Mediterranean Basin
Source: Sci Rep. 2018 Jan 18;8:1078. doi: 10.1038/s41598-018-19446-3 (PMC5773550; doi:10.1038/s41598-018-19446-3)
Supplement: Supplementary file 1 — Supplementary information [file 41598_2018_19446_MOESM1_ESM.pdf]

Supplementary Information for:

Evidence of the Zanclean megaflood in the eastern Mediterranean Basin

Aaron Micallef[1]*, Angelo Camerlenghi[2], Daniel Garcia-Castellanos[3], Daniel Cunarro Otero[1], Marc-André Gutscher[4], Giovanni Barreca[5], Daniele Spatola[1], Lorenzo Facchin[2], Riccardo Geletti[2], Sebastian Krastel[6], Felix Gross[6], Morelia Urlaub[7]

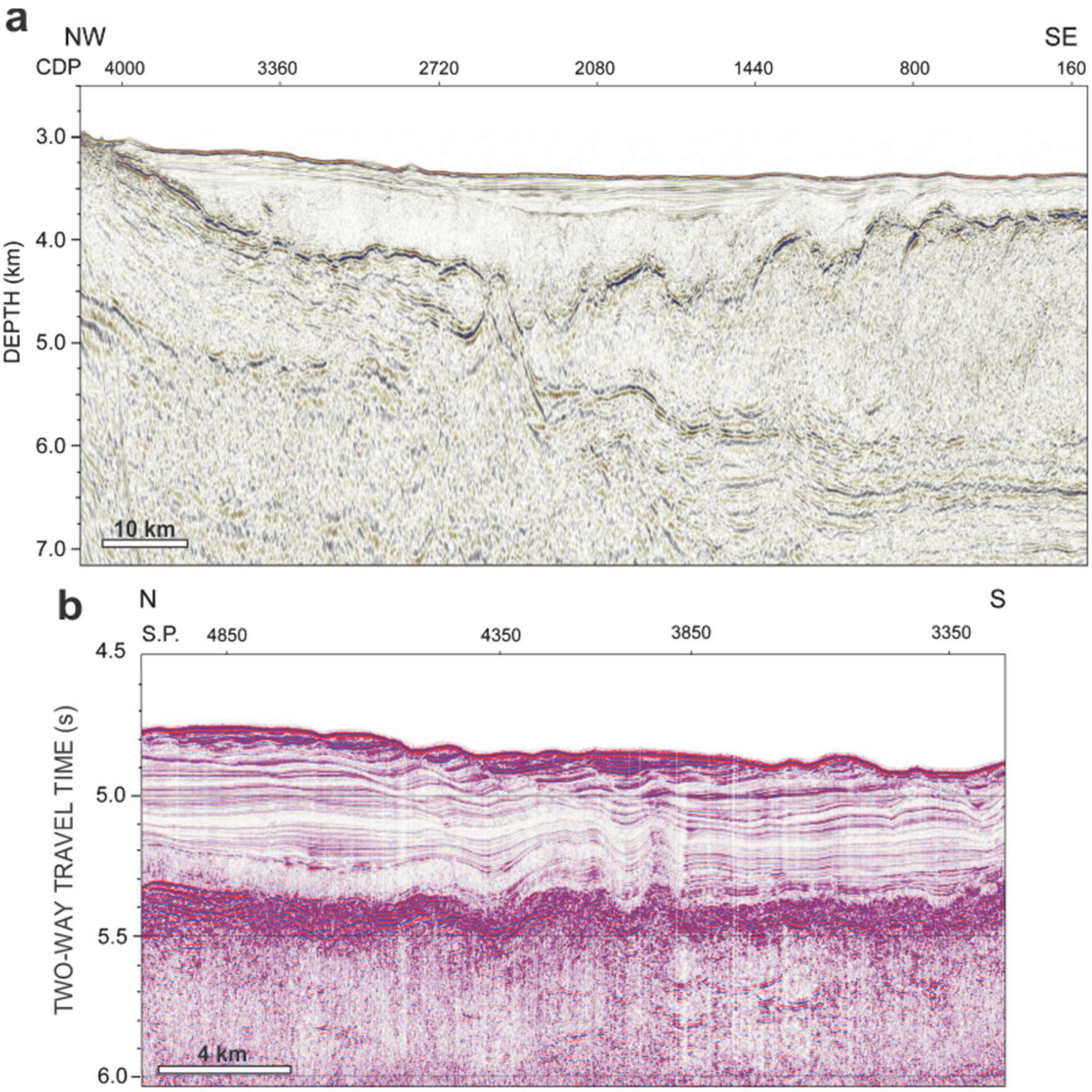

Supplementary Fig. S1: Seismic reflection profiles from western Ionian Basin. Uninterpreted (a) PSDM seismic reflection profile CROP 21 and (b) post-stack time-migrated seismic reflection profile CUMECS-3. Location of profiles in Fig. 2a. CDP = common depth point; SP = shot point.

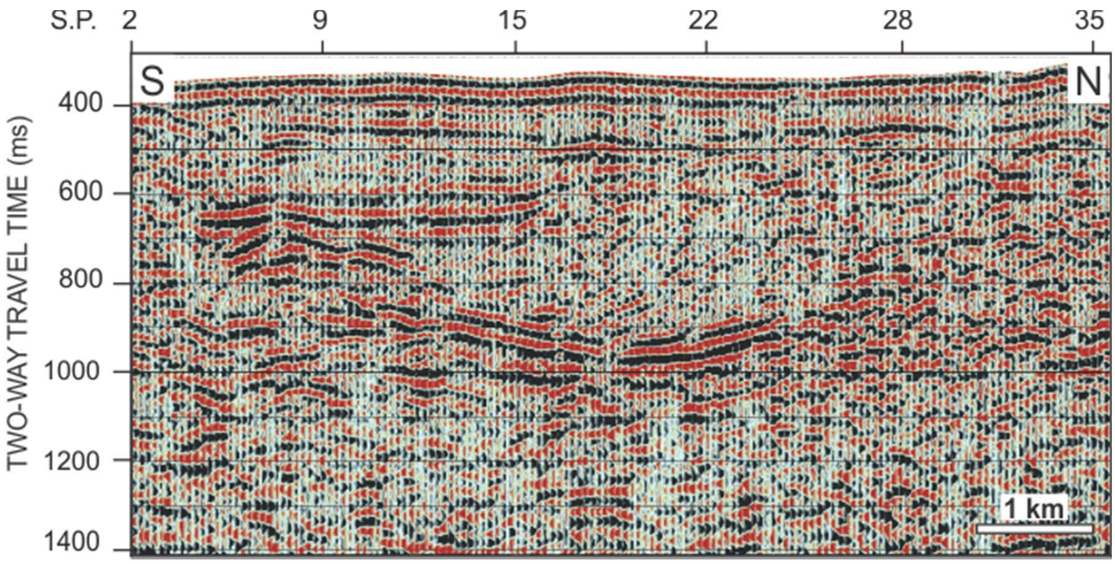

Supplementary Fig. S2: Seismic reflection profile upslope of Noto Canyon.

Uninterpreted seismic reflection profile C-578. Location of profile in Fig. 3a. SP = shot point.

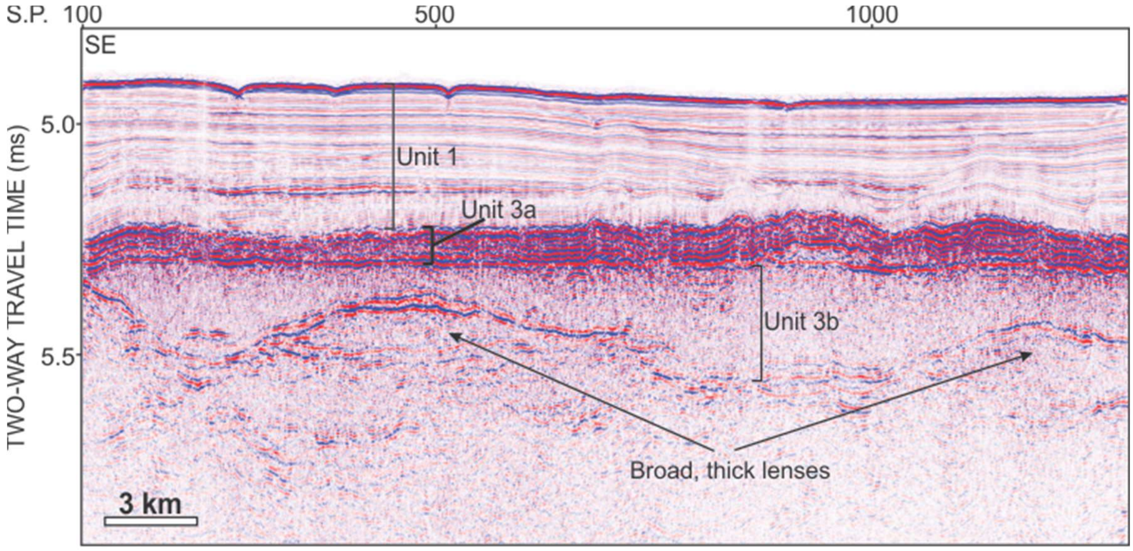

Supplementary Fig. S3: Seismic reflection profile from the southern part of western Ionian Basin. Interpreted seismic reflection profile CUMECS-3 showing broad, thick lenses underneath unit 3b (halite). The salt deposition clearly seals a pre-existing seabed topography controlled by sediment mass transport and deposition. Location of profile in Fig. 3e. Interpretation based on DSDP Site 374. SP = shot point.

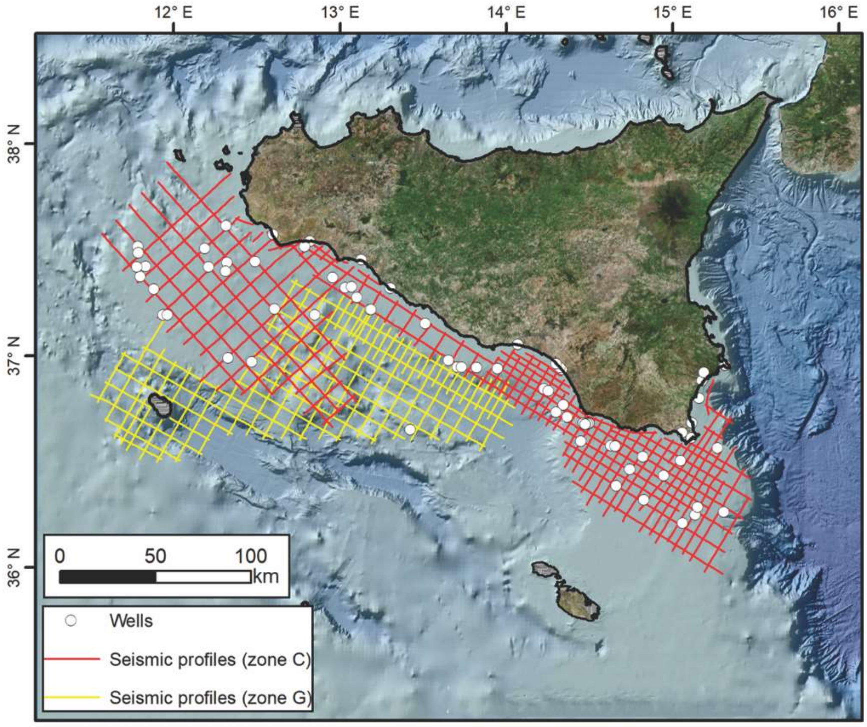

Supplementary Fig. S4: Spatial coverage of seismic reflection profiles and wells from VIDEPI (http://unmig.sviluppoeconomico.gov.it/videpi/; VIDEPI 2009-2017; Ministero dello sviluppo economico DGRME, Società Geologica Italiana, Assomineraria; Creative commons license 3.0: https://creativecommons.org/licenses/by/3.0/). No changes have been made to the original data. Map generated with ArcMap 10.2 (http://www.esri.com/arcgis/). Background data from EMODnet bathymetry (www.emodnet-bathymetry.eu/) and a compilation of data from ESRI, DigitalGlobe, GeoEye, Earthstar Geographics, CNES Airbus DS, USDA, USGS, AeroGRID, and IGN.

Supplementary Table S1: Stratigraphy of the western Ionian Basin. Age, seismic markers and lithology of interpreted units from seismic reflection profiles.

Units	Age	MSC seismic marker; Depositional units [12]	Lithology after DSDP Site 374	Lithology after [12] assuming similar composition to Western Mediterranean units
Unit 1	Quaternary and Pliocene	P-Q	From top down: Nannofossil marl with graded unit of foraminiferal quartzose sand to silt; Nannofossil marl and mud; Nannofossil marl and ooze	-
			Lower Pliocene: Dolomite	Lower Pliocene: Biogenic ooze
Unit 2	Miocene-Pliocene transition (Eastern Mediterranean Zanclean Flood)	Not defined elsewhere	Not present at drill site location	Not defined elsewhere
Unit 3a	Messinian	Upper Unit (UU)	Dolomitic mudstone with minor gypsum layers; Gypsum/dolomitic mudstone cycles; Anhydrite and salts	Anhydrite layers interbedded with marls
Unit 3b		Mobile Unit (MU)	not drilled	Halite
Unit 3c		Lower Unit (LU)	not drilled	Reworked gypsum? Turbidites? Clastics?
